# Comparison of the efficacy and tolerability of telmisartan and enalapril in patients of mild to moderate essential hypertension

**DOI:** 10.4103/0253-7613.66838

**Published:** 2010-06

**Authors:** Pramod B. Akat, Tushar R. Bapat, Mangala B. Murthy, Vitthal B. Karande, Shreyas R. Burute

**Affiliations:** Department of Pharmacology, Government Medical College, Miraj, Maharashtra, India

**Keywords:** Blood pressure, diastolic BP, enalapril, hypertension, systolic BP, telmisartan

## Abstract

**Background:**

Theoretically, angiotensin II receptor blockers (ARBs) have certain advantages over angiotensin-converting enzyme inhibitors, but the contribution of these advantages to the clinical effect of ARBs is not known.

**Objective:**

To compare the efficacy and tolerability of telmisartan with enalapril in patients of essential hypertension.

**Materials and Methods:**

Patients of mild to moderate hypertension were randomized to receive either 40 mg of telmisartan or enalapril 10 mg once a day orally for 12 weeks. At each visit, the systolic blood pressure (BP), diastolic BP and heart rate of each patient were recorded. Investigations such as hemogram hemoglobin, total leucocytes count (Hb, TLC), serum creatinine, serum glutamic oxaloacetic transaminase, serum glutamic pyruric transaminase (SGOT, SGPT) random blood sugar and urine examination were performed at baseline and after 12 weeks of the treatment period.

**Results:**

The mean reduction in systolic BP in the telmisartan/enalapril group was 26.38 ± 10.98/26.74 ± 8.24 mmHg while the mean reduction in diastolic BP in the telmisartan/enalapril group was 14 ± 2.98/9.71 ± 4.23 mmHg, respectively, at 12 weeks. When the reduction in systolic BP in the two groups was compared, there was no significant difference between the groups (*P* > 0.05). However, the mean reduction in diastolic BP achieved with telmisartan at 12 weeks was significantly higher (*P* < 0.001) than that achieved with enalapril after the corresponding period. The overall frequency of adverse-effects was similar. However, in the enalapril group, the incidence of dry cough was higher as compared to that in the telmisartan group (11.43% vs. 0%, respectively; *P* < 0.05).

**Conclusion:**

Telmisartan produces a greater reduction in diastolic BP than enalapril and is free from the adverse-effect of dry cough that is commonly encountered with enalapril.

## Introduction

Hypertension is a “life time” condition and, if left untreated, leads to lethal complications. The rennin–angiotensin system plays an important role in the regulation of normal blood pressure (BP) and also in the pathogenesis and maintenance of essential hypertension.

Currently, two classes of drugs that attenuate the action of angiotensin II and act as antihypertensive agents by different mechanisms are available, which include angiotensin-converting enzyme (ACE) inhibitors and angiotensin II receptor blockers (ARBs). ACE inhibitors act by reducing the biosynthesis of angiotensin II by blocking the action of ACE on angiotensin I but do not inhibit the alternative non-ACE angiotensin II generating pathways whereas ARBs block the AT1 receptor-mediated actions of angiotensin II without inhibiting the synthesis of angiotensin II. Taking into consideration the alternative pathways of angiotensin II generation, ARBs reduce the activation of AT1 receptor more effectively than do ACE inhibitors.[1]

The current place of ARBs in the treatment of essential hypertension is as an alternative to ACE inhibitors in patients who do not tolerate them due to adverse-effects.

But, there is constant debate over the comparative efficacy of ACE inhibitors and ARBs due to the additional advantages of ARBs over ACE inhibitors.[2]

Hence, this study was undertaken to evaluate whether the above-mentioned theoretical benefits actually translate into clinically observable benefits in patients of mild to moderate essential hypertension.

The ACE inhibitor, enalapril, and ARB, telmisartan, were used in the present study.

## Materials and Methods

The study was conducted in the Out-Patient Department of Medicine, Government Medical College, Miraj, after the approval of the Institutional Ethics Committee. This was a prospective, open, randomized, comparative, controlled clinical trial.

A total of 80 patients were enrolled in the study as per the selection criteria. Patients with mild to moderate essential hypertension of either sex within the age group of 18–65 years were included in the study. They were either newly diagnosed patients or were those who had discontinued antihypertensive medication voluntarily for more than 4 weeks.

The following categories of patients were excluded from the study: patients on other antihypertensive therapy, patients of secondary hypertension, patients with impaired liver function defined as SGOT or SGPT >2 times the normal limit, patients with impaired kidney function confirmed by serum creatinine >2 mg/dl, pregnant and lactating females, patients with history suggestive of obstructive biliary disease, cholestasis or severe hepatic impairment and female patients of the child-bearing age group not using medically approved contraceptives.

The patients were enrolled after informed and written consent as per the inclusion and exclusion criteria. Current medical history and diagnosis were noted during the first visit. Patients were randomly assigned to receive either telmisartan or enalapril, with 40 patients in each group.

Group A patients received 40 mg of telmisartan once a day orally for 12 weeks while group B patients received 10 mg of enalapril once a day orally for 12 weeks.

After enrollment into the study, follow-up was performed after 2 weeks, 4 weeks, 8 weeks and 12 weeks. At each visit, complete clinical examination was carried out, including a recording of systolic and diastolic blood pressure (BP) of each patient using a mercury sphygmomanometer by the auscultation method. The BP was recorded in a sitting position after 10 min of rest. The pressure at which the sounds were first heard was taken as the systolic pressure and the pressure at which the sounds disappeared was taken as the diastolic pressure.

The heart rate was also measured at each visit. Investigations such as hemogram (Hb, TLC), serum creatinine, SGOT, SGPT, random blood sugar and urine examination were performed during the first visit and after 12 weeks of the study period.

The primary efficacy end point was the change from baseline sitting diastolic BP. Other end points included change from baseline in systolic BP. Patients who did not complete the full 12 weeks of therapy as per the study regulations were not included for statistical analysis. Safety was assessed in terms of both subjective and objective systemic adverse-effects. Subjective symptoms such as headache, dizziness, fatigue, back pain, dyspepsia, myalgia, pruritus, nausea and dry cough were assessed by questioning the patient at each visit. Objective signs like rash and hypotension were obtained and biochemical examination was carried out.

Qualitative data on adverse-effects were analyzed by using the Z-test for difference between proportions. Quantitative data were analyzed by using the Z-test for difference between means. A *P*-value <0.05 was taken as significant and *P*-value <0.001 was taken as highly significant, while *P* >0.05 was considered as insignificant.

## Results

Baseline clinical characteristics of patients receiving telmisartan and enalapril were compared. The two groups were similar and comparable as regards systolic BP, diastolic BP and heart rate before treatment.

In the telmisartan-treated group, the mean systolic BP prior to treatment was 154.72 ± 12.52 mmHg. After treatment, the systolic BP reduced to 143.16 ± 10.33 mmHg, 138.94 ± 9.47 mmHg, 133.61 ± 8.29 mmHg and 128.33 ± 7.50 mmHg at 2 weeks, 4 weeks, 8 weeks and 12, weeks respectively. The reduction in systolic BP was found to be statistically significant (*P* < 0.001) at 2 weeks, 4 weeks, 8 weeks and 12 weeks of therapy when compared with the baseline readings.

The mean diastolic BP before telmisartan treatment was 98.22 ± 3.78 mmHg. After treatment, the diastolic BP reduced to 90.05 ± 1.47 mmHg, 88.94 ± 2.36 mmHg, 86.44 ± 3.61 mmHg and 84.22 ± 3.78 mmHg at 2 weeks, 4 weeks, 8 weeks and 12 weeks, respectively. The reduction in diastolic BP was found to be statistically significant (*P* < 0.001) at 2 weeks, 4 weeks, 8 weeks and 12 weeks of therapy when compared with the baseline readings.

In the enalapril-treated group, the mean systolic BP prior to treatment was 156.05 ± 10.56 mmHg. After treatment, the systolic BP reduced to 141.6 ± 17.94 mmHg, 139.82 ± 9.37 mmHg, 133.77 ± 8.53 mmHg and 129.31 ± 7.32 mmHg at 2 weeks, 4 weeks, 8 weeks and 12 weeks, respectively. The reduction in the mean systolic BP was found to be statistically significant (*P* < 0.001) at 2 weeks, 4 weeks, 8 weeks and 12 weeks of therapy when compared with the baseline readings.

The mean diastolic BP before enalapril treatment was 98.34 ± 4.45 mmHg. After treatment, the diastolic BP reduced to 90.62 ± 1.66 mmHg, 89.77 ± 1.26 mmHg, 89.37 ± 2.04 mmHg and 88.63 ± 1.35 mmHg at 2 weeks, 4 weeks, 8 weeks and 12 weeks, respectively. The reduction in the diastolic BP with enalapril was found to be statistically significant (*P* < 0.001) at 2 weeks, 4 weeks, 8 weeks and 12 weeks of therapy when compared with the baseline readings.

The mean reduction in systolic BP in the telmisartan/enalapril group was 11.56 ± 6.39/14.45 ± 19.88 mmHg, 15.77 ± 8.65/16.22 ± 6.63 mmHg, 21.11 ± 8.53/22.28 ± 7.39 mmHg and 26.38 ± 10.98/26.74 ± 8.24 mmHg, respectively, at 2 weeks, 4 weeks, 8 weeks and 12 weeks. When the reduction in systolic BP in the two groups was compared, there was no significant difference between the groups (*P* > 0.05).

The mean reductions in diastolic BP in the telmisartan/enalapril group were 8.16 ± 3.32/7.71 ± 3.91 mmHg and 9.27 ± 3.73/8.57 ± 3.93 mmHg, respectively, at 2 weeks and 4 weeks. When the values were compared in both the treatment groups, the difference was not statistically significant (*P* > 0.05).

The mean reductions in diastolic BP in the telmisartan/enalapril groups at 8 weeks and 12 weeks were 11.78 ± 3.34/8.97 ± 3.8 mmHg and 14 ± 2.98/9.71 ± 4.23 mmHg, respectively. The mean reduction in diastolic BP achieved with telmisartan at 8 weeks and 12 weeks was significantly higher (*P* < 0.001) than that achieved with enalapril at the corresponding period.

The safety analysis was performed on all patients who completed the study. A total of 8.33% of the patients reported some sort of adverse-effects like fatigue (2.77%), headache (2.77%) and dizziness (2.77%) in the telmisartan group whereas this figure was 22.85% in the enalapril group, with noted adverse-effects like fatigue (2.85%), headache (2.85%), dizziness (2.85%), nausea (2.85%) and dry cough (11.43%). However, this difference in the frequency of adverse-effects between the groups was not statistically significant (*P* > 0.05). In the enalapril-treated group, the incidence of dry cough was 11.43% while it was 0% in the telmisartan-treated group. When both the groups were compared, the incidence of dry cough was significantly higher in the enalapril group as compared with the telmisartan group (*P* < 0.05).

## Discussion

The rennin–angiotensin system plays a crucial role in regulation of BP. The primary effector peptide angiotensin II functions at two receptors, AT1 and AT2. The AT1 receptor mediates actions like vasoconstriction and aldosterone secretion while the AT2 receptor mediates vasodilatation and natriuresis.

ACE inhibitors reduce biosynthesis of angiotensin II whereas ARBs completely block AT1 receptors, and both are effective antihypertensive agents. But, currently, there is a constant debate over the comparative efficacy of ACE inhibitors and ARBs due to the possibility of angiotensin II generation by alternative pathways with the use of ACE inhibitors. Hence, ARBs are said to reduce the activation of AT1 receptors more effectively than ACE inhibitors. Therefore, this study was undertaken to compare the antihypertensive efficacy of ACE inhibitors with ARBs.

As a prototype and widely used ACE inhibitor, enalapril was taken for comparison with telmisartan, which has special characteristics such as a longer half-life (24-h elimination) and an insurmountable antagonism at angiotensin II receptors. Once-daily telmisartan has been reported to attenuate angiotensin II-induced increases in the diastolic and systolic BP and it is said to have a rapid antihypertensive effect within 3 h of administration of a single oral dose, and BP control is reported to be sustained throughout the 24-h dosage interval, including the last 6 h of the dosage interval.[3]

The two groups were comparable to each other in terms of age, weight and baseline characteristics such as sex ratio, smoking and alcohol habits. 22.5% of the patients in the telmisartan group had received other medications like non-steroidal anti-inflammatory drugs (NSAIDs,) antibiotics and H_2_ blockers temporarily as and when required, whereas this figure was 20% for the enalapril group. These patients were included because treatment of hypertension is life-long and patients often need these drugs temporarily.

In the present study, 32.5% of the patients of the telmisartan group and 37.5% of the patients of the enalapril group were previously on antihypertensive therapy. They had discontinued therapy voluntarily due to economic constraints or other reasons for 4 weeks or more before enrollment into the study. The period of 4 weeks was sufficient to washout the effect of any previous antihypertensive drug.

Thus, in the present study, we have observed that both telmisartan and enalapril are effective agents in reducing both systolic and diastolic BP throughout the study period when measured at the 2^nd^, 4^th^, 8^th^ and 12^th^ week. When efficacy of telmisartan was compared with enalapril, we found that telmisartan was as effective as enalapril in reducing systolic BP [Table 1], but telmisartan is more effective in reducing diastolic BP when compared with enalapril [Table 2].

**Table 1:** Comparative effect of telmisartan and enalapril on systolic blood pressure

*Parameters*	*Telmisartan systolic BP in mmHg (mean ± SD)*	*Enalapril systolic BP in mmHg (mean ± SD)*	*P-value*
Baseline	154.72 ± 12.52	156.05 ± 10.56	>0.05
After 2 weeks	143.16 ± 10.33	141.6 ± 17.94	>0.05
After 4 weeks	138.94 ± 9.47	139.82 ± 9.37	>0.05
After 8 weeks	133.61 ± 8.29	133.77 ± 8.53	>0.05
After 12 weeks	128.33 ± 7.50	129.31 ± 7.32	>0.05

**Table 2:** Comparative effect of telmisartan and enalapril on diastolic blood pressure

*Parameters*	*Telmisartan diastolic BP in mmHg (mean ± SD)*	*Enalapril diastolic BP in mmHg (mean ± SD)*	*P-value*
Baseliner	98.22 ± 3.78	98.34 ± 4.45	>0.05
After 2 weeks	90.05 ± 1.47	90.62 ± 1.66	>0.05
After 4 weeks	88.94 ± 2.36	89.77 ± 1.26	>0.05
After 8 weeks	86.44 ± 3.61	89.37 ± 2.04	<0.001
After 12 weeks	84.22 ± 3.78	88.63 ± 1.35	<0.001

This finding of our study is similar to that of another study[4] in which a significantly greater proportion of patients achieved low values of diastolic BP with telmisartan than with enalapril (59% versus 50%; *P* < 0.05).

In a randomized, double-blind, double-dummy, parallel group study,[5] telmisartan 40 mg produced a significantly greater reduction from baseline diastolic BP compared with enalapril 10 mg (11.7 versus 8.7 mmHg, respectively; *P* = 0.02). In another comparative multicentric trial[6] between telmisartan 80 mg and enalapril 20 mg, the mean reduction in systolic and diastolic BP were significantly higher with telmisartan as compared with enalapril (systolic BP, *P* = 0.013; diastolic BP, *P* = 0.002).

This greater reduction in the mean diastolic BP associated with telmisartan might be due to the selective blockade of the angiotensin II AT1 receptor so that the actions of angiotensin II produced by the alternative pathway are also blocked. This does not happen with enalapril, which inhibits ACE but not the other non-ACE angiotensin II generating pathways.

Secondly, telmisartan selectively blocks only the AT1 receptor and not the AT2 receptors and thus the actions of angiotensin II that are mediated through AT2 receptors are not blocked. AT2 receptor stimulation by angiotensin II causes vasodilatation via bradykinin, nitric oxide and c-GMP, causing natriuresis. Therefore, angiotensin II, by activating the AT2 receptor, produces vasodilatation and natriuresis, which may also contribute to the antihypertensive action of ARBs like telmisartan.

Most ARBs decrease the systolic and diastolic BP significantly as compared to baseline values but most studies utilizing telmisartan have shown a favorable decrease in the diastolic BP over and above the ACE inhibitor. This might not be a class effect of ARBs as some studies utilizing other ARBs like losartan and valsartan have not shown such effects.[7] The characteristic effect of telmisartan in decreasing the diastolic BP may be related to its long half-life (24-h elimination) as other ARBs with shorter half-lives may activate compensatory mechanisms like sympathetic stimulation, which compromises their vasodilatory effect. No serious adverse-effect necessitating stoppage of therapy was encountered in any patient receiving telmisartan. The adverse-events encountered with telmisartan were mild and transient, like headache, dizziness and fatigue. The incidence of these adverse-effects was similar to that of enalapril. However, in the enalapril group, 11.43% of the patients complained of dry cough while this adverse-effect was not noticed in any of the patients receiving telmisartan.

Both telmisartan and enalapril did not produce any adverse impact on various parameters like hemoglobin, TLC, serum creatinine, SGOT, SGPT and random blood sugar. This again indicates a good safety profile of telmisartan.

Thus, it can be concluded that telmisartan is as effective as enalapril in reducing systolic BP but produces a greater reduction in diastolic BP than enalapril and is free from the adverse-effect of dry cough, which is commonly encountered with enalapril.

Thus, because of its efficacy throughout 24 h and because of a better tolerability, telmisartan could be preferred as a first-line drug among the ARBs for the treatment of mild to moderate essential hypertension.

One of the striking differences between previous studies comparing ACE inhibitors and ARBs and the present study is the significantly greater reduction in diastolic BP with telmisartan as compared with enalapril. However, there is strong evidence that systolic BP is a better predictor of cardiovascular events among elderly as compared with diastolic BP.[8] The role of diastolic BP as a predictor of cardiovascular complications is currently under debate. Hence, further studies are required to substantiate any long-term beneficial effect of telmisartan in patients with high diastolic BP.
